# Dr. Louis A. Duhring

**Published:** 1913-09

**Authors:** 


The late Dr. Louis A. Duhring of Philadelphia, emeritus professor of dermatology in the University of Pennsylvania, left most of his million dollar estate to Philadelphia charities and philanthropies, including a bequest to the University.

